# Confirmatory factor analysis for two questionnaires of caregiving in eating disorders

**DOI:** 10.1080/21642850.2014.894889

**Published:** 2014-03-19

**Authors:** Rebecca Hibbs, Charlotte Rhind, Hannah Sallis, Elizabeth Goddard, Simone Raenker, Agnes Ayton, Bryony Bamford, Jon Arcelus, Nicky Boughton, Frances Connan, Ken Goss, Bert Lazlo, John Morgan, Kim Moore, David Robertson, Christa Schreiber-Kounine, Sonu Sharma, Linette Whitehead, Hubert Lacey, Ulrike Schmidt, Janet Treasure

**Affiliations:** ^a^ Eating Disorders Unit, Institute of Psychiatry, King's College London, London, UK; ^b^ MRC Integrative Epidemiology Unit, University of Bristol, Bristol, UK; ^c^ The Darwin Centre, North Staffordshire Combined Healthcare NHS Trust, London, UK; ^d^ Adult Eating Disorders Service, South West London and St George's NHS Trust, London, UK; ^e^ Eating Disorders Service, Leicestershire Partnership NHS Trust, Leicester, UK; ^f^ Cotswold House Eating Disorders Service, Oxford Health NHS Foundation Trust, Oxford, UK; ^g^ Vincent Square Eating Disorders Service, Central and North West London Mental Health NHS Trust, London, UK; ^h^ Coventry Eating Disorder Service, Coventry and Warwickshire NHS Partnership Trust, Coventry, UK; ^i^ Haldon Unit, Devon Partnership NHS Trust, Exeter, UK; ^j^ Yorkshire Centre for Eating Disorders, Leeds and St George's University of London, London, UK; ^k^ Eating Disorders, South Staffordshire and Shropshire NHS Foundation Trust, Stafford, UK; ^l^ National Centre for Mental Health, Birmingham and Solihull Mental Health NHS Foundation Trust, Birmingham, UK; ^m^ STEPS Eating Disorders Unit, Avon and Wiltshire Partnership Mental Health NHS Trust, Bristol, UK; ^n^ Eating Disorders Service, The Priory Hospital Cheadle Royal, Manchester, UK; ^o^ Eating Disorders Research Team, St George's, University of London, London, UK

**Keywords:** caregiving, eating disorders, confirmatory factor analysis, burden, accommodation and enabling

## Abstract

Objective: Caring for someone diagnosed with an eating disorder (ED) is associated with a high level of burden and psychological distress which can inadvertently contribute to the maintenance of the illness. The Eating Disorders Symptom Impact Scale (EDSIS) and Accommodation and Enabling Scale for Eating Disorders (AESED) are self-report scales to assess elements of caregiving theorised to contribute to the maintenance of an ED. Further validation and confirmation of the factor structures for these scales are necessary for rigorous evaluation of complex interventions which target these modifiable elements of caregiving. Method: EDSIS and AESED data from 268 carers of people with anorexia nervosa (AN), recruited from consecutive admissions to 15 UK inpatient or day patient hospital units, were subjected to confirmatory factor analysis to test model fit by applying the existing factor structures: (a) four-factor structure for the EDSIS and (b) five-factor structure for the AESED. Results: Confirmatory factor analytic results support the existing four-factor and five-factor structures for the EDSIS and the AESED, respectively. Discussion: The present findings provide further validation of the EDSIS and the AESED as tools to assess modifiable elements of caregiving for someone with an ED.

## Introduction

Caring for a loved one diagnosed with an eating disorder (ED) is associated with a high level of burden and psychological distress (Dimitropoulos, Carter, Schachter, & Woodside, 2008; Treasure et al., 2008; Zabala, MacDonald, & Treasure, 2009). The interpersonal maintenance model, which can be applied trans-diagnostically, describes a causal chain whereby high levels of carer unmet needs (Graap et al., 2008; Haigh & Treasure, 2003) deplete coping resources (Coomber & King, 2012) and contribute to carer anxiety and depression. In turn, carers exhibit high expressed emotion (e.g. emotional over-involvement, criticism and hostility) and ineffective strategies in managing symptoms (e.g. accommodating and enabling behaviours). These responses may inadvertently allow the ED to flourish (Treasure & Schmidt, 2013). A vicious cycle is set in motion whereby carer anxiety is mirrored by the sufferer which in turn exacerbates illness symptoms (Goddard, Salerno, et al., 2013). Empirical testing is essential for rigorous complex intervention evaluation and for further refinement of the underpinning theoretical framework. The interpersonal maintenance model provides a theoretical basis for interventions which can target modifiable elements of caregiving. Skills-based approaches involving psycho-education and problem-solving skills groups for families of people with anorexia nervosa (AN) have reduced family distress and emotional over-involvement and have led to an improvement in the ED behaviours (Holtkamp, Herpertz-Dahlmann, Vloet, & Hagenah, 2005; Sepulveda, Lopez, MacDonald, & Treasure, 2008; Uehara, Kawashima, Goto, Tasaki, & Someya, 2001; Vandereycken & Louwies, 2005; Whitney et al., 2012; Zucker, Ferriter, Best, & Brantley, 2005). Self-management tools for carers (book and DVDs) (Treasure, Smith, & Crane, 2007) that specifically target maintaining factors reduced carer distress, expressed emotion and accommodating and enabling behaviours (Goddard, MacDonald, Sepulveda, et al., 2011). For further rigorous evaluation of interventions which target response to illness, outlined by the interpersonal maintenance model, well-validated scales which measure modifiable elements of caregiving are necessary.

The Eating Disorders Symptom Impact Scale (EDSIS) (Sepulveda, Whitney, Hankins, & Treasure, 2008) was developed to measure the range of symptoms (namely nutrition, guilt, social isolation and dysregulated behaviour) which have a direct impact on carers of people with EDs. The Accommodation and Enabling Scale for Eating Disorders (AESED) (Sepulveda, Kyraciou, & Treasure, 2009) measures caregivers' behaviours which inadvertently serve to reinforce or fail to discourage symptoms or behaviours. For example, symptomatic behaviours such as high control over family food and meal rituals may go unchecked (accommodating) and negative consequences of behaviour (e.g. clearing up bathroom mess following a purge) are not applied (enabling) (Sepulveda et al., 2009).

Both scales have demonstrated satisfactory psychometric properties in their initial validation in a community sample of carers, with EDSIS factors and AESED subscales explaining 58.5% and 60.1% of the variance in carer distress, respectively. Good reliability was reported for the EDSIS (Cronbach's alpha ranging from 0.84 to 0.90) and the AESED (Cronbach's alpha ranging from 0.77 to 0.92). Moreover, moderate correlations were found with specific elements hypothesised to contribute to illness maintenance included in other measures associated with caregiving such as the Experience of Caregiving Inventory (Szmukler et al., 1996), the Hospital Anxiety and Depression Scale (Zigmond & Snaith, 1983) and the Levels of Expressed Emotion (LEE) (Cole & Kazarian, 1988).

The original four-factor structure of the EDSIS has been used in carer intervention outcome studies (Goddard, MacDonald, Sepulveda, et al., 2011; Grover, Naumann, et al., 2011; Grover, Williams, et al., 2011; Hoyle, Slater, Williams, Schmidt, & Wade, 2013; Pepin & King, 2013; Sepulveda, Lopez, Todd, Whitaker, & Treasure, 2008) as has the original five-factor structure of the AESED (Goddard, MacDonald, Sepulveda, et al., 2011). However, a six-factor structure for the EDSIS has since been proposed within an Australian sample of carers of someone with an ED (Coomber & King, 2013). In their sample, AN carers reported significantly lower level binge-purge impacts compared with bulimia nervosa (BN) carers, and a significantly higher level of mealtime difficulties than BN carers. Given the broad and complex range of symptoms and behaviours associated across EDs and their severity, and the associated different manifestations of caregiver burden and response to illness, further research examining the factor structure of the EDSIS and AESED in a single and stable diagnosis cohort is warranted.

The aim of the present study then, is to use confirmatory factor analysis (CFA) to investigate how well the factor structure of the EDSIS and AESED fits data from a sample of 268 carers of people with severe and enduring AN admitted for hospital treatment. The size of the sample should enable robust reassessment of scales, including possible item exclusion and factor structure examination for overall and subscale scores.

## Method

### Design

Data were collected as part of the baseline assessment of a multi-centre randomised controlled trial evaluating a skills-based intervention for carers of someone with AN (Carer, Assessment, Skills and Information Sharing (CASIS)) (Goddard, Raenker, et al., 2013). Ethical approval was granted by the Royal Free Hospital Ethics Committee (CREC ref no. 08/H0720/41).

### Sample

Carers (*n* = 268) of people diagnosed with AN were recruited as part of the CASIS study. Patients admitted to 15 UK inpatient or day patient units with a primary diagnosis of AN or Eating disorder not otherwise specified with anorexic symptoms (EDNOS-AN) were offered the opportunity to participate in the research and at least one carer, identified by the patient, had to participate for the family to be included in the study. The final sample consisted of 144 mothers, 81 fathers and 28 partners (Table 1). There were also eight siblings, five friends and two “other relatives” in the sample. The majority of carers were white (95.8%), employed (63.9%) and parents of the sufferer (83.9%). Most mothers and partners described themselves as primary carers (97.2% and 92.9%, respectively), whereas the majority of fathers described themselves as secondary carers (92.6%). Definition of primary and secondary carer was subjective but was related to the level of dependence by the patient and number of hours of contact. The patients were all admitted to National Health Service (NHS) specialist ED services at the time of data collection and written consent to contact their carers was obtained from them by clinicians or clinical studies officer. After receiving the patients' consent, carers were contacted by the researchers and written consent was obtained. Inclusion criteria for participants required individuals to be fluent in English and able to provide consent. Patients had to have a primary diagnosis of AN or EDNOS-AN and have at least one carer consent to participate in the project. Patients were excluded if they or their carers were taking part in another treatment study. Both patients and carers had to be aged 12 years or older and participants with an identified severe comorbidity (e.g. severe learning disability and psychosis) were also excluded. All participants (patients and carers) completed self-report assessments by post at admission to the treatment hospital.

**Table 1.** Demographic and clinical data of carers and service users.

Variable	Service user (*n* = 178)	Carer		
		Mother (*n* = 144)	Father (*n* = 81)	Partner (*n* = 28)
	M (SD)	M (SD)	M (SD)	M (SD)
Age^a^	26.0 (9.0)	53.3 (7.3)	54.9 (8.6)	39.3 (12.1)
	*N* (%)	*N* (%)	*N* (%)	*N* (%)
*Gender*				
Female	169 (94.9)	144 (100)	0	2 (7.1)
Male	9 (5.1)	0	81 (100)	26 (92.9)
*Education*				
No qualification	9 (5.2)	13 (9.3)	4 (5.1)	2 (7.1)
O/A-levels	89 (51.4)	48 (34.3)	25 (31.7)	9 (32.1)
University/higher degree	73 (42.2)	64 (45.7)	37 (46.8)	17 (60.7)
Other	2 (1.2)	15 (10.7)	13 (16.5)	0
Missing	5	4	2	0
*Employment*				
Paid employed – full-time	16 (9.2)	41 (28.9)	52 (65.8)	16 (59.3)
Paid employed – part-time	10 (5.7)	44 (31.0)	3 (3.8)	5 (18.5)
Homemaker/unemployed/sick/retired	95 (54.6)	57 (40.1)	24 (30.4)	4 (14.8)
Student	53 (30.5)	0	0	2 (7.4)
Missing	4	2	2	1
*Marital status*				
Married/living together	35 (20.3)	111 (77.6)	72 (91.1)	22 (78.6)
Single/divorced/widowed	137 (79.7)	32 (22.4)	7 (8.9)	6 (21.4)
Missing	6	1	2	0
*Carer type*				
Primary carer	–	140 (97.2)	6 (7.4)	26 (92.9)
Secondary carer	–	4 (2.8)	75 (92.6)	2 (7.1)
*Living with patient prior to hospitalisation*				
Yes	–	95 (66.4)	50 (62.5)	22 (78.6)
No	–	48 (33.6)	30 (37.5)	6 (21.4)
Missing	–	1	1	0

Notes: M, mean and SD, standard deviation.

^a^1 service user with missing age.

### The Eating Disorders Symptom Impact Scale (Sepulveda, Whitney, et al., 2008)

The EDSIS is a 24-item self-report measure of caregiving burden in EDs. The scale is tailored to a population of ED carers and comprises subjective and objective burden. The scale has high internal consistency (Cronbach's alpha = 0.91) across four subscales managing nutritional situations, guilt, dealing with dysregulated behaviours and social isolation. Scores are obtained on a five-point Likert scale. Higher scores indicate higher caregiving burden and more negative appraisal of caregiving.

### The Accommodation and Enabling Scale for Eating Disorders (Sepulveda et al., 2009)

The AESED is a 33-item self-report measure used to assess the degree of accommodating and enabling behaviours to the ED. A five-point Likert scale is used to yield a total score and subscale scores including: avoidance and modifying routine; reassurance seeking; meal ritual; control of family and turning a blind eye. This scale has good internal consistency (Cronbach's alpha = 0.76).

### Statistical analysis

SPSS Version 18 and Amos Version 20 were used for the analysis. The characteristics of the sample were summarised and predictors of missing data were assessed. Due to the lack of independence between carers, logistic regression using a robust variance estimator was used to look at the distribution of missing data.

A CFA was carried out by applying the factor structure as described in the original papers detailing each questionnaire (Sepulveda et al., 2009; Sepulveda, Whitney, et al., 2008). In order to account for missing responses to items, a maximum likelihood approach to the analysis was used. This method does not delete cases or impute missing observations but estimates parameters and their standard errors directly from the available data (Kline, 2010).

Given that the sample consisted of both primary and secondary carers of the same sufferers and therefore not independent, CFA was not applied across the entire sample. Instead, carers were divided into groups according to the primary or secondary carer status and the factor model was applied to these groups separately to assess measurement invariance. The same analysis was run comparing male and female carers to check that the assessment of measurement invariance was not biased. The outcome was the same. Measurement invariance gauges whether scores in the two carer groups have the same meaning and can be fitted with the same factor structure (Kline, 2010). The stronger the measurement invariance, the more parameters are assumed to be equal in both primary and secondary carers. A weak invariance model builds on the simplest form of measurement invariance, configural invariance, in which only the number of factors and their associated indicators are assumed to be the same between groups, by constraining the unstandardised factor loadings to be equal in both groups. Constraining the means of the primary and secondary carers to be equal would constitute strong invariance. The strongest form of measurement invariance is that of strict invariance. Under this model, all parameters are assumed to be the same for both primary and secondary carers. That is, all carers are constrained to have an identical factor model specification with equal factor loadings, correlations, means and residual variances. Residual variance is the item variance not explained by the factor (Kline, 2010; Wu, Shen, & Bruno, 2007).

A chi-squared test was used to assess model fit and a non-significant result (*p *> .05) indicates a good model fit. When comparing between invariance models, the same threshold (*p* > .05) was used. Where the factors contained items which were highly correlated, or items that did not load on the latent variable, an additional exploratory analysis looked at removing some of these items in an attempt to improve the fit of the model. Where several items on the same factor had standardised regression weights of 0.80 or above, one or more of these were considered for removal from the model. The decision as to which item(s) to remove was based on expert opinion resulting from discussion among the study team.

The following measures were used to assess the model fit: the chi-square (*χ* ^2^) statistic and degrees of freedom (DF); relative *χ* ^2^ (normed chi-square = *χ* ^2^/DF) which is the chi-squared statistic divided by the DF of the model where values of less than 2.5 indicate a good model fit (Carmines & McIver, 1981); comparative fit index (CFI), which measures the proportion of covariation in the data that can be reproduced by a given model where values of greater than 0.9 represent a good model fit; and root mean square error of approximation (RMSEA), which is the discrepancy per DF. An excellent model fit is indicated by RMSEA values lower than 0.05 while a close model fit is suggested by values between 0.05 and 0.08 (Hoyle, 2011).

## Results

There were missing data in response to both questionnaires. Eighty-seven percent of people returned complete data on all items of the EDSIS, and this was slightly lower for the AESED with 79% of respondents completing all items. In part, this was due to a printing error which resulted in missing responses to items 1–10 for a minority of carers (*n* = 14, 5% of sample). Secondary carers were less likely to complete the entire questionnaire in comparison to primary carers (EDSIS: 1.7% vs. 5.7%, respectively, and AESED: 1.7% vs. 7.8%, respectively). This resulted in a final sample size of 260 for the EDSIS and 258 for the AESED.

The Cronbach's alpha for both scales was high. Reliability of each subscale of the AESED (avoidance and modifying routine = 0.89, reassurance seeking = 0.88, meal ritual = 0.89, control of family = 0.87 and turning a blind eye = 0.83) in addition to the overall reliability (alpha = 0.93) were high. The EDSIS also showed good reliability overall (alpha = 0.87) and for each of the subscales (nutrition = 0.83, guilt = 0.87, dysregulated behaviour = 0.73 and social isolation = 0.83).

### Factor analysis – EDSIS

A four-factor structure had previously been suggested for this 24-item questionnaire (Sepulveda, Whitney, et al., 2008), with items corresponding to the following factors: nutrition (8 items), guilt (5 items), dysregulated behaviour (7 items) and social isolation (4 items).

Multiple group CFA was conducted between primary and secondary carers to assess measurement invariance. The model of configural invariance was shown to be an acceptable fit to the data (RMSEA = 0.070 (90% CI: 0.065–0.075); CFI = 0.77; Chi square/degree of freedom ratio = 2.30). However, a comparison with the weak invariance model showed this stronger form of measurement invariance to be the better fit to the data (*p* = .608). The weak invariance model again demonstrated an acceptable model fit (RMSEA = 0.068 (90% CI: 0.063, 0.073); CFI = 0.77; *χ*2 = 1156 (DF = 518); *χ*2/DF = 2.23). Comparison with both the strong and the strict invariance models did not show any improvement on the fit (both *p* < .001) and so the weak invariance model was retained.

Both unstandardised and standardised regression coefficients for this model are shown in Table 2, while between-factor covariances and correlations are reported in Table 3. Different standardised results are shown for primary and secondary carers; this is due to the fact that within the weak invariance model, equal variances have not been imposed on the two groups.

**Table 2.** EDSIS questionnaire – weak invariance model, both unstandardised and standardised results are shown.

	Unstandardisedestimate	S.E.	*p*	Standardisedestimates	
				Primarycarers	Secondarycarers
*Factor 1: Nutrition*					
18. Did you spend a long period of time shopping for food	1.0	–	–	0.574	0.615
22. Did you check on her to ensure that she/he was ok	0.656	0.098	<.001	0.548	0.590
23. Did you notice or think about how the illness was affecting her/him physically	0.946	0.139	<.001	0.601	0.591
15. Were there arguments with other family members about how to handle mealtimes	1.095	0.141	<.001	0.632	0.695
24. Did you notice or think about how the illness was affecting her/him mentally	0.697	0.096	<.001	0.632	0.635
21. Did you have to turn up the heat due to her/him feeling cold	0.949	0.14	<.001	0.536	0.560
16. Were there arguments or tension during mealtimes	1.198	0.142	<.001	0.697	0.774
14. Did you experience difficulties preparing meals	1.263	0.148	<.001	0.722	0.683
*Factor 2: Guilt*					
8. Thinking that perhaps I was not strict enough	1.0	–	–	0.436	0.523
5. Feeling that I should have noticed it before it became so bad	1.471	0.205	<.001	0.670	0.718
9. Thinking about where I went wrong	1.621	0.208	<.001	0.833	0.862
7. Feeling that there could have been something I should have done	1.818	0.226	<.001	0.937	0.943
6. Feeling that I have let her/him down	1.835	0.232	<.001	0.880	0.941
*Factor 3: Dysregulated behaviour*					
11. Controlling/manipulative	1.0	–	–	0.853	0.742
20. Were there bad smells and poor hygiene in the bathroom	0.279	0.099	.005	0.210	0.186
17. Did food disappear from the cupboards	0.388	0.115	<.001	0.247	0.219
10. Physically and/or verbally aggressive	0.821	0.073	<.001	0.761	0.620
13. Out of control temper	0.769	0.072	<.001	0.702	0.705
12. Lying/stealing	0.563	0.058	<.001	0.643	0.565
19. Did you have difficulties with blocked drains, plumbing	0.249	0.08	.002	0.230	0.208
*Factor 4: Social isolation*					
3. Feeling unable to go out for evenings, weekends or on holiday	1.0	–	–	0.744	0.749
4. Cancelling or refusing plans to see friends or relations	0.930	0.081	<.001	0.758	0.763
2. Losing your friends	0.865	0.103	<.001	0.722	0.747
1. How your friends/relatives have stopped visiting	0.979	0.117	<.001	0.735	0.728

Note: Different standardised results are shown for primary and secondary carers since equal variances have not been imposed on the two groups.

**Table 3.** EDSIS – weak invariance model, both unstandardised (covariance) and standardised (correlation) results displayed.

			Covariance			Correlation	
			Estimate	S.E.	*p*	Primarycarers	Secondarycarers
Nutrition	<–>	Guilt	.110	.035	.002	.278	.235
Nutrition	<–>	Dysregulated behaviour	.226	.053	<.001	.367	.403
Nutrition	<–>	Social isolation	.273	.06	<.001	.459	.421
Social isolation	<–>	Guilt	.133	.039	<.001	.289	.286
Social isolation	<–>	Dysregulated behaviour	.283	.059	<.001	.396	.506
Guilt	<–>	Dysregulated behaviour	.154	.039	<.001	.323	.383

Within this model, most items were shown to correlate highly with their assigned factors, giving results similar to the original factor analysis. The exception to this was the dysregulated behaviour factor, for which three of the standardised regression coefficients were all found to be lower than 0.25. This suggests that these items (17, 19 and 20*)* did not contribute a great deal to the factor and that the model fit could potentially be improved by removing these items. However, since each of these items relate to different symptoms (missing food, plumbing problems and bad hygiene, respectively), after discussion with the study team it was decided that, on balance, these items would not be eliminated. Additionally, several items (6, 7 and 9*)* on the guilt factor were found to be highly correlated. After discussion among the study team, it was decided to remove item 7 (“Feeling that there could have been something that I should have done”) given that the content of this item overlaps with, and is better elicited by, item 6 (“Feeling that I have let her/him down”) and 9 (“Thinking about where I went wrong”).

Although removing this item resulted in a very slight improvement of the model fit, the weak invariance model was still found to be the best fit to the data (RMSEA = 0.063 (90% CI: 0.056, 0.070); CFI = 0.82; *χ*2 = 719 (DF = 350); *χ*2/DF = 2.06).

### Factor analysis – AESED

A five-factor model was previously suggested for this questionnaire consisting of 33 items (Sepulveda et al., 2009). The following factors were suggested: avoidance and modifying routine (10 items), reassurance seeking (8 items), meal ritual (7 items), control of family (4 items) and turning a blind eye (4 items).

Multiple group CFA was again carried out to assess measurement invariance between primary and secondary carers. In this instance, strict invariance was found to hold. In both primary and secondary carers the factor loadings, between-factor correlations, intercepts and residual variances can be assumed equal. The strict invariance model was found to be superior to the configural invariance (*p* = .334), weak invariance (*p* = .120) and strong invariance (*p* = .169) models. The strict invariance model was found to be an adequate fit to the data (RMSEA = 0.062 (90% CI: 0.059–0.066); CFI = 0.78; *χ*2 = 2197 (DF = 1079); *χ*2/DF = 2.04). Unstandardised and standardised regression coefficients are shown in Table 4 and between-factor covariances and correlations are shown in Table 5.

**Table 4.** AESED questionnaire – strict invariance model, both unstandardised and standardised results are shown.

	UnstandardisedEstimate	S.E.	*p*	Standardisedestimate
*Factor 1: Avoidance and modifying routine*				
25. How often did you participate in behaviours related to your relative's compulsions over the last week	1.0	–	–	0.534
24. To what extent would you say that the relative with an ED controls family life and activities	0.521	0.074	<.001	0.576
26. How often did you assess your relative in avoiding things that might make him/her anxious	0.868	0.125	<.001	0.565
33. Has your relation become angry/abusive when you have not provided assistance	1.207	0.161	<.001	0.635
29. Have you modified your work schedule because of your relative's needs	1.062	0.151	<.001	0.578
32. Has your relative become distressed when you have not provided assistance	1.430	0.171	<.001	0.772
27. Have you avoided doing things, going places or being with people because of your relative's disorder	1.301	0.16	<.001	0.732
30. Have you modified your leisure activities because of your relative's needs	1.291	0.159	<.001	0.725
28. Have you modified your family routine because of your relative's symptoms	1.315	0.158	<.001	0.769
31. Has helping your relative in the previously mentioned ways caused you distress	1.313	0.156	<.001	0.784
*Factor 2: Reassurance seeking*				
8. Repeated conversations about ingredients and amounts in food preparation	1.0	–	–	0.730
10. Repeated conversations about self-harm	0.381	0.063	<.001	0.401
9. Repeated conversations about negative thoughts and feelings	0.989	0.088	<.001	0.740
18. Accommodation of routines of checking their body shape or weight	0.889	0.097	<.001	0.602
6. Repeated questioning whether it is safe or acceptable to eat certain foods	1.179	0.094	<.001	0.820
5. Repeated questioning about whether she will get fat	1.265	0.094	<.001	0.878
7. Repeated seeking of reassurance about whether she looks fat in certain clothes	1.215	0.094	<.001	0.843
17. Accommodation of the exercise routine of the relative with an ED	0.674	0.097	<.001	0.463
*Factor 3: Meal Ritual*				
13. Accommodating to what time food is eaten	1.0	–	–	0.543
19. Accommodating to how the house is cleaned and tidied	1.306	0.151	<.001	0.758
14. Accommodating to what place food is eaten in	1.063	0.142	<.001	0.597
16. Accommodating to how food is stored	1.491	0.157	<.001	0.913
11. Accommodating to what crockery is used	1.184	0.143	<.001	0.695
12. Accommodating to how crockery is cleaned	1.120	0.129	<.001	0.765
15. Accommodating to how the kitchen is cleaned	1.520	0.159	<.001	0.932
*Factor 4: Control of Family*				
1. Control choice of food that you buy	1.0	–	–	0.762
2. Control what family members do and for how long in the kitchen	1.144	0.092	<.001	0.799
4. Control what other family members eat	1.065	0.09	<.001	0.766
3. Control cooking practice and ingredients used	1.213	0.09	<.001	0.868
*Factor 5: Turning a blind eye*				
21. Ignore if money is taken	1.0	–	–	0.530
22. Ignore kitchen left in a mess	3.296	0.386	<.001	0.891
20. Ignore food disappearing	2.652	0.348	<.001	0.690
23. Ignore bathroom left in a mess	3.326	0.392	<.001	0.871

**Table 5.** AESED questionnaire – strict invariance model, both unstandardised (covariance) and standardised (correlation) results displayed.

			Covariance			Correlation
			Estimate	S.E.	*p*	Estimate
Avoidance and modifying routine	<–>	Control of family	.383	.07	<.001	.575
Avoidance and modifying routine	<–>	Meal ritual	.259	.056	<.001	.466
Avoidance and modifying routine	<–>	Turning a blind eye	.073	.019	<.001	.35
Avoidance and modifying routine	<–>	Reassurance seeking	.322	.064	<.001	.476
Control of family	<–>	Meal ritual	.364	.073	<.001	.463
Control of family	<–>	Turning a blind eye	.080	.024	<.001	.271
Control of family	<–>	Reassurance seeking	.426	.081	<.001	.445
Meal ritual	<–>	Turning a blind eye	.038	.018	.037	.154
Meal ritual	<–>	Reassurance seeking	.324	.07	<.001	.407
Reassurance seeking	<–>	Turning a blind eye	.058	.023	.012	.192

Within this model, most items again demonstrated a good level of association with their assigned factor and results were generally similar to those of the original factor analysis. Within four of the factors, there were several items which correlated very highly: meal ritual (15 and 16), control of family (2 and 3), reassurance seeking (5, 6 and 7) and turning a blind eye (22 and 23). After discussion among the study team, item 7 (“Does your relative engage any family member in repeated conversations asking for reassurance about whether she/he looks fat in certain clothes”) was excluded from the model because it was thought to be made redundant by item 5 (“Does your relative engage any family member in repeated conversations asking for reassurance about whether she/he will get fat?”), whereas items 15, 16, 2, 3 and 6 were thought to relate to separate behaviours.

Using the new factor structure, strict invariance was once again found to hold. However, with the exclusion of just one item, the model fit remained very similar to that of the initial model specification (RMSEA = 0.061 (90% CI: 0.057, 0.065); CFI = 0.79; *χ*2 = 2006 (DF = 1014); *χ*2/DF = 1.98).

## Discussion

The aim of the study was to reassess the factor structure of the EDSIS and AESED in a single and stable diagnosis cohort of carers. The size of the sample enables robust reassessment of scales, including possible item exclusion and factor structure examination for overall and subscale scores. Results from the CFA provide support for the validity of the existing four-factor structure for the EDSIS and the five-factor structure for the AESED for carers of a severe and enduring AN patient group. Multiple group CFA examined the stability of these factor structures across primary and secondary carers. Standards of weak invariance for the EDSIS and strict invariance for the AESED were achieved by supporting the robustness of the factor structure and the internal reliability for the items and subscales irrespective of the carer status. Exceptions were for a few highly correlated items on both scales and for items in the EDSIS which did not contribute significantly to the model. Reanalysis of the model fit omitting item 7 of the EDSIS and item 7 of the AESED very slightly improved the model fit in both cases. Due to sampling variation, there is always a chance that various items will not fit the data as well and since the original analysis found these items to be important in the factor structure, and the model fit is only marginally improved by removing it in either case, the justification for removal is not enough to warrant revised versions of either questionnaire.

Although differences in the EDSIS overall and in subscale scores between primary and secondary carers have previously been reported (Sepulveda et al., 2012), the findings of the present study suggest that the EDSIS and the AESED are robust scales in measuring modifiable elements of caregiving across carers. Second, the present study provides the largest dataset of carers completing the EDSIS and the AESED available to date.

As already described, unmet carer needs can lead to high LEE and to accommodating and enabling behaviours which contribute to the maintenance of the illness and consequently poorer prognosis (Goddard, MacDonald, Sepulveda, et al., 2011). The examination of these elements of caregiving is essential to further the discussion of the cognitive interpersonal maintenance model (Treasure & Schmidt, 2013; Treasure, Sepulveda, et al., 2007) and for the development of carer interventions for which valid and reliable measures which are sensitive to change are crucial. There is evidence to suggest that elements of caregiving are modifiable following skills-based carer interventions with improved sufferer outcomes (Goddard, MacDonald, & Treasure, 2011) and the results of this study provide further validation of two measures as tools to assess these elements in a homogenous sample of carers of someone admitted for inpatient care with AN. From a clinical perspective, well-validated tools to assess caregiving enable robust assessment of the specific difficulties faced by ED carers and results from this study and those of Coomber and King (2013), suggest that specific difficulties may be dependent on illness presentation and duration. This is important for caregiving interventions targeting relevant behaviours. We hope that this research will pave the way for interventions that have the potential to improve outcomes in severe and enduring AN at a small cost to the NHS.

### Study limitations and recommendations for future research

The AESED is designed for carers who live with the sufferer. When this is not the case (e.g. during hospital admission), or the behaviour in question is not present, an item response “0” may reflect an absence of this behaviour rather than a true representation of levels of accommodating and enabling. Therefore caution should be taken when interpreting the questionnaire. Having said this, the carers in our sample completed the questionnaires when the patient was in the hospital (i.e. not at home) and the AESED was still valid according to the CFA.

The small percentage of missing data, particularly from the AESED, should be considered in interpreting the results. There were no systematic differences between completed and missing data groups on demographic variables, with the exception of primary/secondary carer status, with missing questionnaires more frequent among secondary carers.

Multiple group CFA examining the stability of the EDSIS and AESED factors across carers of adolescents compared with adult sufferers may provide direction for further refinement of the theoretical model of caregiving.

## Conclusions

Overall, the present findings provide further validation of the EDSIS and the AESED as tools to assess modifiable elements of caregiving for someone with an ED.
